# LOXL2 as a protective in osteoarthritis cartilage

**DOI:** 10.18632/aging.101317

**Published:** 2017-10-27

**Authors:** Manish V. Bais, Mary B. Goldring

**Affiliations:** Department of Molecular and Cell Biology, Boston University Henry M. Goldman School of Dental Medicine, Boston, MA 02118, USA

**Keywords:** LOXL2, osteoarthritis, cartilage regeneration

While osteoarthritis (OA) affects the older population leading to disability with progressive age (65+), there remain few therapeutic options. Identifying agents that protect against cartilage damage could provide breakthroughs for disease prevention and treatment. However, there is no anabolic agent approved for clinical application. We recently implicated a copper-dependent amine oxidase, lysyl oxidase like-2 (LOXL2), as a potential protective factor in OA cartilage that could lead to cartilage regeneration. LOXL2 has known roles in extracellular matrix (ECM) remodeling and collagen crosslinking. We discovered that LOXL2 expression is increased during endochondral ossification in a fracture healing model in mice [1]. LOXL2 function is also critical for normal chondrogenic differentiation [2]. More recently, we have uncovered potentially novel functions of LOXL2. LOXL2 is expressed in the degenerative lesions of cartilage in OA-affected knee-, hip- and temporo-mandibular joints (TMJ) as part of a compensatory anabolic response [3].

Our studies demonstrate that ectopic LOXL2 expression in human OA articular chondrocytes (HAC-OA) induces anabolic gene expression [COL2A1, SOX9, ACAN, chondroitin sulfate proteoglycan 4 (CSPG4, lubricin)], while attenuating catabolic genes [matrix metalloproteinase 13 (MMP13) and NF-κB signaling] in vitro and in vivo [3]. Human chondrocytes embedded in Matrigel from knee joints (HAC-OA/Matrigel) or from temporomandibular joints (TMJ-OA/Matrigel) and implanted in nude mice become myogenic over time in vivo, but this phenotype is prevented by Adv-LOXL2 injection [3]. LOXL2 transduction results in down-regulation of phospho-SMAD2/3 showing in these implants that LOXL2 could inhibit or provide feedback inhibition to TGFβ1-induced signaling [3]. Gene set enrichment analysis showed that transduction of HAC-OA with LOXL2 enriched biosynthesis and proteo-glycan networks [3]. Therefore, the LOXL2 has the potential to open new therapeutic avenues for this debilitating disease.

**Figure 1:**
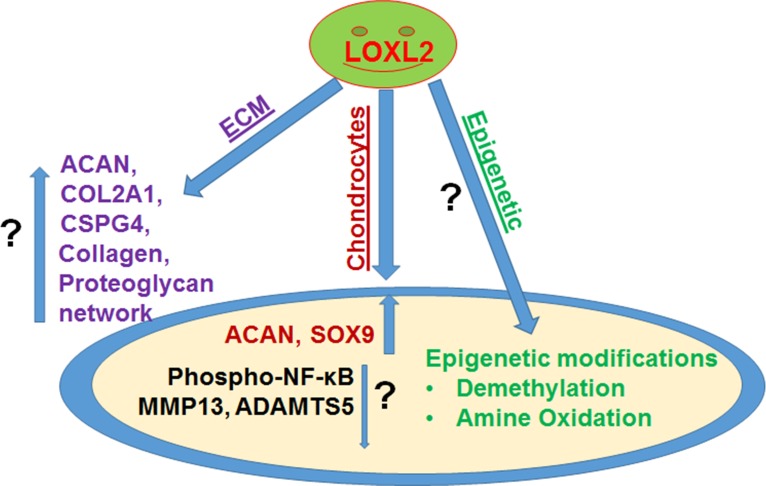
The role of LOXL2 in osteoarthritis and cartilage regeneration Our studies showed that LOXL2 transduction in vitro and in vivo induces anabolic gene expression [COL2A1, SOX9, ACAN, lysine-specific demethylase 6B (KDM6B), chondroitin sulfate proteoglycan 4 (CSPG4, lubricin)], extracellular matrix (ECM) remodeling and proteoglycan deposition, while attenuating catabolic genes [matrix metalloproteinase 13 (MMP13) and NF-κB signaling]. However, LOXL2 induced specific mechanism and epigenetic regulation during anabolic response are yet to be characterized in details. Increased and decreased level by arrows (up-down) and unknown role (?) are indicated in figure.

LOXL2 has extracellular and intracellular functions. During OA, collagen levels decline due to degenerative changes; however, LOXL2 is a collagen crosslinking enzyme that enables collagen deposition and resistance to proteolysis. Although LOXL2 has extracellular functions in the ECM, it also perinuclear region, and cytoplasm and co-localizes with SOX9 in the nucleus. Intracellularly, it could function in transcriptional regulation and epigenetic changes. Nuclear LOXL2 could induce direct epigenetic changes [4] or act indirectly through methylation/demethylation regulators acting on histones binding to promoter, leading to their transcriptional activation of chondro-genic lineage genes. These epigenetic regulators are involved in transcriptional regulation and epigenetic changes, in different cellular contexts [5]. Alternatively, nuclear LOXL2 could induce oxidation and demethy-lation of a lysine residue on specific histone proteins due to its amine oxidation function. Thus, we contend that LOXL2 has specific anabolic functions in OA (Figure 1).

Indeed, our studies were the first to propose a role for LOXL2 in OA pathophysiology. However, the precise mechanisms underlying the roles of LOXL2 in vivo in normal cartilage development, maintenance, and protection in OA cartilage are not known. LOXL2 could induce a chondroprotective response through inhibition of inflammation- or stress-induced signaling pathways or an unknown epigenetic mechanism(s). We have, therefore, taken the opportunity to obtain insight into a novel genetic and epigenetic function for LOXL2 in OA. One potential mechanism is that defective LOXL2 function on its substrate (collagen) could lead to OA is not known. Structural modifications in collagen could alter the substrate specificity of LOXL2 function, which needs to be evaluated by genetic and epigenetic studies. This concept is supported by studies which showed that mutation of *Col11a1* in the chondrodysplasia mouse [6] or *Col9a1* deficiency in mice leads to age-dependent OA and increase susceptibility to cartilage degradation [7].

Taken together, these results led us to ask the following questions (Figure 1): Does the loss of LOXL2 induce an OA-related pro-inflammatory response? Does nuclear LOXL2 enzyme have a specific epigenetic function? Does LOXL2 exert a protective function in normal and OA cartilage, and if so, can it be exploited in pre-clinical models to uncover new therapeutic strategies for OA? Our long-term objectives are to understand LOXL2 induced molecular mechanism of OA etiology and pathogenesis and identify its therapeutic potential for OA.

LOXL2 may have broader implications for knee and TMJ cartilage regenerative therapies. In the United States, an annual cost estimated for treating TMJ disorders including OA is $4 billion, as published by NIDCR, and costs for knee OA, including joint replacement exceed $185 billion. Although fibroblast growth factor (FGF) 18 and bone morphogenetic protein (BMP) 7 proteins and IL-1 antagonists are in phase 1 and 2 clinical trials for OA, LOXL2 could provide better therapeutic potential due to its ability to promote a specific anabolic response in OA. LOXL2 is a naturally acting enzyme that has both chondro-protective and anabolic effects in OA [3]. The potential of LOXL2 to serve as a novel candidate OA drug should be evaluated in progressive aging and OA preclinical models for future clinical applications.
